# Implicit bias in digital health: systematic biases in large language models’ representation of global public health attitudes and challenges to health equity

**DOI:** 10.3389/fpubh.2025.1705082

**Published:** 2025-11-28

**Authors:** Yuan Gao, Yican Feng, Surng Gahb Jahng

**Affiliations:** 1 Advanced Graduate School of Imaging, Chung-Ang University, Seoul, Republic of Korea; 2 Fashion Design Institute, Zhejiang Fashion Institute of Technology, Ningbo, China

**Keywords:** large language models, representational bias, digital health, health equity, algorithmic audit

## Abstract

**Introduction:**

As emerging instruments in digital health, large language models (LLMs) assimilate values and attitudes from human-generated data, thereby possessing the latent capacity to reflect public health perspectives. This study investigates into the representational biases of LLMs through the lens of health equity. We propose and empirically validate a three-dimensional explanatory framework encompassing Data Resources, Opinion Distribution, and Prompt Language, positing that prompts are not just communicative media but critical conduits that embed cultural context.

**Methods:**

Utilizing a selection of prominent LLMs from the United States and China-namely Gemini 2.5 Pro, GPT-5, DeepSeek-V3, and Qwen 3. We conduct a systematic empirical analysis of their performance in representing health attitudes across diverse nations and demographic strata.

**Results:**

Our findings demonstrate that: first, the accessibility of data resources is a primary determinant of an LLM’s representational fidelity for internet users and nations with high internet penetration. Second, a greater consensus in public health opinion correlates with an increased propensity for the models to replicate the dominant viewpoint. Third, a significant “native language association” is observed, wherein Gemini 2.5 Pro and DeepSeek-V3 exhibit superior performance when prompted in their respective native languages. Conversely, models with enhanced multilingual proficiencies, such as GPT-5.0 and Qwen 3, display greater cross-lingual consistency.

**Discussion:**

This paper not only quantifies the degree to which these leading LLMs reflect public health attitudes but also furnishes a robust analytical pathway for dissecting the underlying mechanisms of their representational biases. These findings bear profound implications for the advancement of health equity in the artificial intelligence era.

## 1 Introduction

We are currently situated at the nexus of a paradigm shift in health, one driven by data and artificial intelligence (AI). Large Language Models (LLMs), as revolutionary informational media and interactive instruments, are permeating the public health domain with unprecedented depth and breadth. This heralds a new era of digital health characterized by greater intelligence, precision, and personalization, with the potential to fundamentally reshape public health interventions and personalized medicine (1). Contemporary models such as Google’s Gemini 2.5 Pro, OpenAI’s GPT-5, and China’s DeepSeek V3 and Qwen 3, leveraging their formidable capabilities in natural language understanding and generation, exhibit a profound potential to redefine global health practices. Their capacity to function as proxies for human respondents, replicating findings from complex social science research, offers a novel methodological avenue for exploring public health attitudes (2). However, this potential is accompanied by significant challenges. The realization of their promise hinges on a critical, yet largely unexamined, presupposition: that LLMs can accurately and impartially comprehend and reflect the diverse health attitudes, cultural beliefs, and core concerns of global populations.

When LLMs function as a novel “public opinion infrastructure” capable of direct dialog with billions of users, a scrupulous examination of their intrinsic value orientations and latent biases becomes imperative (3). LLMs do not create knowledge ex nihilo; rather, they learn linguistic patterns, factual knowledge, and indeed, societal biases from vast corpora of pre-training data (4). The challenge of bias in artificial intelligence is complex and multifaceted, permeating every stage of the model lifecycle. For instance, in its authoritative report, the National Institute of Standards and Technology (NIST) categorizes the sources of bias into three main contexts: systemic bias, computational and statistical bias, and human bias (5). Adopting a unique health equity perspective, this study focuses on the representational biases within LLMs, a category of bias that is deeply rooted at the intersection of these three contexts. This learning process implies that the models’ outputs-whether seemingly objective health advice or simulations of public attitudes-are fundamentally probabilistic reproductions of their training data’s distribution (6). If this training data harbors systemic biases-for instance, an overrepresentation of perspectives from high-income, highly educated, and high-internet-penetration nations and populations-then LLMs built upon this foundation risk becoming instruments that amplify, rather than ameliorate, health inequities.

This risk is particularly acute in the domain of health risk perception. Foundational research in behavioral economics and health psychology has long established the prevalence of a cognitive tendency known as “optimistic bias” or “unrealistic optimism” (7, 8). This refers to an individual’s inclination to believe they are less susceptible to negative health events and more likely to experience positive health outcomes compared to their peers (9). Such a bias constitutes a critical psychological barrier, leading individuals to neglect crucial preventative behaviors such as disease screening (10) and vaccination (11), while concurrently encouraging the adoption of high-risk behaviors such as smoking and unhealthy diets (12, 13). It forms the micro-level psychological basis of the “knowledge-action gap” in public health (14). A substantial body of empirical evidence confirms that optimistic bias is widespread across diverse cultures and age groups (15, 16), manifesting with particular salience when confronting significant health threats like cancer (17).

This context gives rise to a set of crucial research questions: How does an LLM respond when confronted with a query concerning health risk perception? Or does it, having been trained on extensive scientific literature, produce a purely “rational,” unbiased answer? Furthermore, do its simulations of health risk perception exhibit systematic variations across different demographic groups (e.g., by gender, age, nationality, or educational attainment)? The answers to these questions bear directly on the reliability, safety, and equity of LLMs in global health applications. Research on LLM bias, while a burgeoning field, has predominantly focused on political leanings (18) and social stereotypes (19), leaving a significant lacuna concerning the specific and highly sensitive domain of public health attitudes. Concurrently, while the field of health psychology offers a wealth of research on risk perception, its subject has invariably been human, failing to incorporate LLMs as new, influential actors in the global health information ecosystem. We identify critical gaps in the current literature: a dearth of specialized research on systemic bias in LLMs’ reflections of global public health attitudes; the absence of a theoretical framework to explain such representational disparities; and a reliance on outdated models, with a lack of large-scale, cross-cultural, and cross-group comparative analyses.

To address these lacunae, this study undertakes a pioneering investigation to systematically evaluate the capacity of the latest generation of US and Chinese LLMs to reflect public health attitudes on a global scale. The core innovation of this research lies not only in describing and quantifying biases but, more importantly, in constructing and proposing a three-dimensional explanatory framework designed to fundamentally uncover the mechanisms influencing the representational fidelity of LLMs. This framework comprises: (1) Data Resource Accessibility, where we hypothesize that the health attitudes of digitally active groups and nations are more accurately captured due to their greater representation in training data, a concept closely linked to the “digital health divide” (20, 21); (2) Opinion Consensus, where we posit that LLMs can more easily reproduce high-consensus public health viewpoints, a notion related to how information environments shape collective opinion (22, 23); and (3) Prompt Language and Cultural Context, where we hypothesize that performance varies systematically with prompt language due to the linguistic composition of training data, analogous to the “language-of-interview effect” (24, 25).

Grounded in this three-dimensional framework, this study will focus on examining the “default bias” of large language models under non-specific contextual prompts. We posit that this baseline state best reflects the information received by ordinary users in real-world application scenarios. This research will employ the large-scale, cross-national World Values Survey (WVS) as a gold standard to systematically test the latest models, including Gemini 2.5 Pro, GPT-5, DeepSeek V3, and Qwen 3. We will quantify the discrepancies between model outputs and the true attitude distributions of various nations and demographic groups based on key social determinants of health (e.g., age, gender, education, and income) (26–28), while also testing the explanatory power of our framework. This research seeks to answer the following core questions: To what extent do the latest LLMs reflect global public health attitudes? Do they exhibit a systemic optimistic bias? Which national and demographic groups are more accurately represented, and can these disparities be explained by the dimensions of data, opinion, and language? The contributions of this study are manifold. Theoretically, it integrates risk perception theory from health psychology (29) with media bias theory from communication studies, offering a novel analytical lens for understanding health information dynamics in the AI era. Practically, our findings will provide crucial empirical evidence for AI developers, public health organizations, and global governance bodies. This aids in the development of more equitable, inclusive, and culturally sensitive AI health applications, ensuring that the dividends of digital technology serve to narrow, rather than widen, existing health disparities.

## 2 Theoretical framework

The outputs of Large Language Models (LLMs) are not objective reflections of reality but are profoundly shaped by the core mechanisms of their training and architecture. To move beyond a mere description of potential biases toward a systematic explanation of their origins, we propose a three-dimensional explanatory framework. This framework is grounded in a macro-level understanding of how bias originates in AI systems. The NIST framework (2022), for example, offers a widely accepted taxonomy that traces bias to data, algorithms, and human-computer interaction. Our proposed “Data Resources-Opinion Distribution-Prompt Language” framework can be seen as an operationalization of these broader concepts within the specific context of LLMs and public health attitudes. For instance, our ‘Data Resources’ dimension directly mirrors the data-driven biases identified by NIST, which are often direct manifestations of deep-seated societal inequities. Furthermore, this framework posits that a fidelity of an LLM’s representation of public health attitudes is contingent upon three key dimensions: the accessibility of data resources, the distribution of opinion on health issues, and the linguistic and cultural context of the interaction. Examination of these dimensions allows for the systematic deconstruction of the pathways through which biases emerge and the formulation of testable hypotheses.

### 2.1 Data resource accessibility and the digital health divide

An LLM’s worldview is fundamentally determined by its pre-training data corpus. Consequently, its capacity to reflect a group’s attitudes is contingent upon the prevalence and accessibility of data representing that group’s perspectives within this corpus (6). In the public health domain, the vast majority of these data are sourced from the public internet, which introduces a significant sampling bias known as the global “digital health divide” (20). This issue manifests at three levels:

First, at the individual level, a stark dichotomy exists between internet users and non-users. The health concerns, experiences, and opinions of internet users are directly harvestable as digital traces from social media and health forums. In contrast, the health attitudes of non-users-who are often older, have lower incomes, or reside in remote areas-are largely absent from this digital ecosystem. Their perspectives enter the training data only indirectly, if at all, via intermediaries such as news media, resulting in “omitted voices in big data” (21). This disparity implies that LLMs are trained on skewed samples of public health concerns, leading to a systematic underestimation of the health realities of offline and socioeconomically disadvantaged populations (30).

Second, at the national level, this disparity is magnified. Nations with high internet penetration rates generate a disproportionate volume of digital text, which in turn disproportionately shapes the data landscape upon which LLMs are trained. Conversely, the public health attitudes of populations in nations with lower connectivity are systematically underrepresented, potentially leading models to generalize the health norms of a few digitally dominant countries to the rest of the world.

Third, at the demographic level, engagement with digital health resources is not uniform even among internet users. Individuals with a higher socioeconomic status (SES) are more likely to generate digital content related to health (31). Consequently, their opinions may be overrepresented. This is particularly critical in public health, as policymaking has already been shown to be biased toward the preferences of high-income groups (26). If LLMs replicate this bias, they risk amplifying the health perspectives of privileged groups while further marginalizing those of vulnerable populations (32). Based on this reasoning, we propose the following hypotheses:

*H1a*: LLMs will represent the health attitudes of internet users with greater fidelity than those of non-users.

*H1b*: The fidelity of an LLM's representation of public health attitudes will be positively correlated with a nation's internet penetration rate.

*H1c*: LLMs will represent the health attitudes of groups with higher educational attainment and income levels with greater fidelity.

### 2.2 Opinion distribution of health issues

Beyond data accessibility, the statistical properties of public opinion on a given health issue affect an LLM’s ability to learn and reproduce it. As probabilistic systems, LLMs are optimized to predict high-probability word sequences. When a strong societal consensus exists on a health topic, the resulting low-entropy information environment provides a clear and robust signal for the model to learn and replicate (33).

However, many critical public health issues are characterized by controversy and a diversity of beliefs. In such high-entropy information environments, the probabilistic signals are weaker and more ambiguous. The model’s capacity to mirror such nuanced diversity is diminished, and it may instead regress toward a simplified or “flattened” representation, potentially amplifying a viewpoint that is only marginally more prevalent in its training data (34). This tendency is analogous to how traditional media can obscure public division by creating a false sense of consensus. Furthermore, online information exposure patterns, whether incidental or selective, can shape the perception of collective opinion (35). Therefore, the greater the real-world consensus on a health issue, the higher the expected fidelity of an LLM’s representation. This leads to our second hypothesis:

*H2*: The fidelity of an LLM’s representation of a public health attitude will be positively correlated with the degree of public consensus on that issue.

### 2.3 Prompt language and cultural barriers

The third dimension of our framework concerns the linguistic and cultural context in which an LLM is prompted. The performance of multilingual LLMs is not uniform across languages but is contingent upon the linguistic composition of their training corpora (36). Given that a substantial portion of the data used to train leading global LLMs is in English, these models develop a more nuanced statistical understanding of English-language concepts. This creates a potential “native language advantage,” whereby the model’s fidelity is superior in its dominant training language.

This phenomenon is analogous to the “language-of-interview effect” observed in survey methodology, where the language of administration can activate different cultural frameworks and influence responses (37). Similarly, prompting an LLM in different languages may activate different subsets of its training data and their associated cultural contexts. This effect is particularly pronounced in a domain health, which is deeply embedded in cultural beliefs and practices (38). An LLM may struggle to represent a culturally specific health attitude if it must map the concept from a less-represented language onto its English-dominated knowledge base. We therefore anticipate that model performance will vary with the alignment between prompt language and the model’s primary training data. Accordingly, we propose our final hypotheses:

*H3a*: US-developed LLMs will represent public health attitudes with greater fidelity when prompted in English compared to Chinese.

*H3b*: Chinese-developed LLMs will represent public health attitudes with greater fidelity when prompted in Chinese compared to English.

## 3 Methods

### 3.1 Analytical benchmark: survey data

To assess representational biases in LLMs, a reliable, cross-national benchmark dataset is required. Here, we employ the World Values Survey (WVS) for this purpose. The WVS is a widely utilized dataset in comparative social science, and its rigorous sampling methodology and standardized questionnaire design provide a robust reference for our investigation (39, 40).

The WVS focuses on deep-seated values that profoundly influence how individuals assess health risks, perceive medical technologies, and view public health policies (41, 42). These stable values constitute the foundation of public health attitudes. Using the WVS as a benchmark therefore enables an evaluation of whether LLMs can capture the structural preferences of human health-related cognitions rather than merely reproducing ephemeral discussions from social media. Furthermore, the WVS includes numerous items related to the social determinants of health, such as attitudes toward family, education, social trust, and technology (43), making it an ideal source of proxy data for a broad spectrum of public health attitudes.

The WVS is conducted in waves, and its sixth wave included questions on internet usage. This allows for the effective differentiation between internet users and non-users, enabling a direct test of the digital divide’s impact on representational fidelity. Therefore, this study utilizes data from the seventh (2017–2022) of the WVS, retaining attitudinal questions common across all surveyed countries. After excluding sensitive items with excessively high refusal rates, we finalized a set of 42 questions for analysis.

### 3.2 Model selection, prompt design, and experimental procedure

To comprehensively assess the representational biases of leading US and Chinese Large Language Models (LLMs), this study selected four influential models. The GPT and Gemini series were chosen as representatives of globally leading models, which are frequently used as performance benchmarks. DeepSeek V3 and Qwen 3 were selected as representatives of leading Chinese models, emblematic of China’s LLM ecosystem due to their consistent iteration and high performance in Chinese.

Therefore, this study selected Gemini 2.5 Pro, GPT-5.0, DeepSeek-V3, and Qwen 3 as its subjects of investigation. We accessed the latest available versions of each model (as of May 2024) via their official API platforms. To systematically examine linguistic preferences, we tested each model using both Chinese and English prompts, and assessed the robustness of our conclusions by setting different temperature parameters. Specific model information and experimental parameters are detailed in Table 1.

**Table 1:** Large language models and core parameters used in the experiment.

Attribute		Google Gemini 2.5 Pro	OpenAI GPT-5.0	DeepSeek-V3	Qwen3-insruct
Developer/country		Google/United States	OpenAI/United States	DeepSeek/China	Alibaba/China
API identifier		Gemini-2.5-Pro-latest	GPT-5.0-turbo-preview	DeepSeek-chat	Qwen3-instruct
Knowledge cutoff		End of 2024	End of 2024	End of 2024	End of 2024
Supported languages		Multilingual (English-optimized)		Multilingual (Chinese-optimized)	
Prompt languages tested		Chinese, English	Chinese, English	Chinese, English	Chinese, English
Temperature Parameter	Low	0.1	0.1	0.1	0.1
	Mid	0.7	1.0	1.0	0.8
	High	1.0	1.5	1.5	1.5
Other parameters		Default values recommended by each model’s API platform were used			

**Figure 1:**
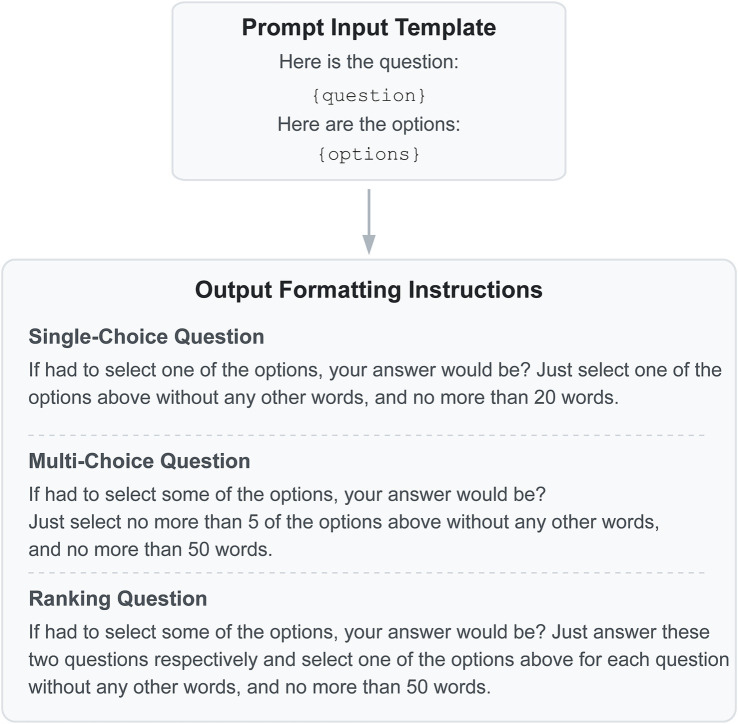
Prompt message process. For Ranking Questions, the prompt includes two sub-questions: “Consider the most important?” and “Consider the next most important?”.

As illustrated in Figure 1, to simulate authentic health survey scenarios, we presented the models with single-choice, multiple-choice, and ranking. The experimental procedure strictly followed the WVS protocols; for instance, we did not provide evasive options such as “do not know” or “refuse to answer,” thereby compelling the models to make a judgment among the given choices. Our Chinese and English prompts were designed and calibrated based on the official WVS questionnaires to ensure cross-lingual consistency. Crucially, to test the models’ inherent biases in their baseline state, the prompts contained no demographic information or specific contextual details about a hypothetical respondent.

As LLMs are fundamentally probabilistic language models, a single response can exhibit stochasticity. This variation, however, stems from the model’s learned internal probability distribution and represents controllable output variance rather than uncontrolled random error (44). To capture a model’s overall disposition, rather than a single stochastic output, we adopted the methodology of prior research (2) and requested 100 independent responses for each survey question, from which we compiled the frequency distribution of selected options. During testing, we excluded items with a refusal rate higher than 10%, which typically involved highly sensitive bioethical topics (45), resulting in a final set of 42 questions for analysis. To process the 100 responses collected for each question, we implemented a highly automated extraction pipeline. This pipeline utilized keyword matching and regular expressions to precisely identify selected option labels from the model outputs. Due to the stringent formatting requirements specified in our prompts (e.g., “Just select one of the options above without any other words”), the vast majority of responses (over 98%) adhered to the requested format and were parsed unambiguously by our script. For the minimal subset of responses (under 2%) containing extraneous text, our script could still reliably extract the core option choice. Cases requiring manual adjudication were exceedingly rare (well under 1% of the total corpus) and had a negligible impact on the final frequency distributions. Consequently, the fidelity of our data processing relies on the accuracy of this automated procedure, obviating the need for a large-scale manual annotation effort.

### 3.3 Variable measurement

The independent variables were designed to operationalize the three-dimensional framework and test the corresponding hypotheses. The first dimension, Data Resource Accessibility (H1a, H1b, H1c), was measured by Digital Access, classifying World Values Survey (WVS) respondents as internet users or non-users. At the national level, we used internet penetration rates from the World Bank for the corresponding survey years (46). Socio-demographics were categorized based on WVS data: age (under 50 versus 50 and over), educational attainment (below university versus university and above), and self-reported income (low, middle, high).

The second dimension, Opinion Distribution (H2), was operationalized as Opinion Concentration, calculated as the standard deviation of the selection proportions for each option within a given demographic group. Higher values indicate greater attitudinal consensus.

The third dimension, Prompt Language and Cultural Context (H3a, H3b), was defined by the Prompt Language (English or Chinese). A country’s official language was included as a control variable in regression models to isolate confounding effects.

The dependent variable was Representational Fidelity, which quantifies the congruence between an LLM’s output distribution and the empirical survey data. Following prior work (6), we measured this using the 1-Jensen-Shannon (JS) distance (47). For each survey question, we calculated the JS distance between two probability distributions: the empirical distribution of a specific human population in the WVS and the distribution generated by an LLM across 100 independent responses. To ensure comparability across different question types, all response data were standardized into probability distributions. For single-choice questions, we calculated the direct frequency of each option. For multiple-choice questions, we computed the relative frequency at which each option was mentioned. Furthermore, to address the zero-frequency issue inherent in such calculations, we applied a standard smoothing technique by adding a small constant, α, to each option’s count before normalization, thereby ensuring computational robustness. The resulting metric, 1-JS distance, ranges from 0 to 1, where a value closer to 1 signifies higher fidelity. A model’s overall representational fidelity for a specific group was calculated by averaging this score across all 42 questions (detailed in the Supplementary material).

### 3.4 Statistical analysis

This study aims to empirically validate the three-dimensional explanatory framework-“Data Resources, Opinion Distribution, and Prompt Language”-proposed in Section 2. We hereby elaborate on the concept and procedures of validation within the context of our research. For complex AI systems like LLMs, framework validation is not about exhaustively confirming or falsifying the framework. Rather, it involves a series of systematic, replicable empirical tests to assess its effectiveness, consistency, and predictive power in explaining the target phenomenon, namely, LLM representational bias. Our validation procedure follows a rigorous cycle of theoretical framework, testable hypotheses, and empirical data analysis.

Specifically, our multi-level statistical strategy directly corresponds to the framework’s three dimensions. First, to test the Data Resources dimension (corresponding to H1a, H1b, H1c), we conduct analyses at both individual and national levels. At the individual level (H1a, H1c), as the distribution of representational fidelity scores violates the assumption of normality, we employ the non-parametric Wilcoxon signed-rank test for paired comparisons across different social groups (e.g., internet users vs. non-users; different levels of education, income, and age). Crucially, to control for the inflated risk of false positives from multiple comparisons across 42 survey items, we apply the BH procedure to all *p*-values for False Discovery Rate (FDR) correction.

At the national level (H1b), we construct linear regression models to examine the effect of national internet penetration (independent variable) on a model’s overall representational fidelity (dependent variable). We acknowledge that the small sample size at this level (N = 10) warrants interpreting these results as exploratory; thus, our focus is on the direction and confidence intervals of the effects rather than precise *p*-values.

Second, to test the Opinion Distribution dimension (H2), we conduct the analysis at the question level. For each survey item, a separate linear regression model is constructed to assess whether public opinion concentration in each country (independent variable) significantly predicts the model’s representational fidelity for that item (dependent variable).

Third, to test the Prompt Language dimension (H3a, H3b), we again use the Wilcoxon signed-rank test, supplemented by regression analysis, to perform paired comparisons of fidelity scores between Chinese and English prompts for the same model.

To ensure the robustness of our findings, control variables, such as a country’s official language, are included in all regression models to account for potential confounders. If this series of tests consistently supports the framework-derived hypotheses across various models and conditions, it would provide strong empirical evidence for the explanatory validity and initial efficacy of our three-dimensional framework, establishing it as a valuable tool for explaining the origins of representational bias in LLMs.

## 4 Results

To quantify the similarity between the LLM output distribution and the real-world survey results from a specific group, this study employed the Jensen-Shannon (JS) distance. As a metric for measuring the divergence between two probability distributions, a smaller JS distance represents less information loss and a better fit (38). Therefore, we used 1-JS distance as the score for representational fidelity; a higher score signifies that the model’s output is more congruent with the health-related attitude distribution of the target population, and thus its representational accuracy is higher.

**Figure 2:**
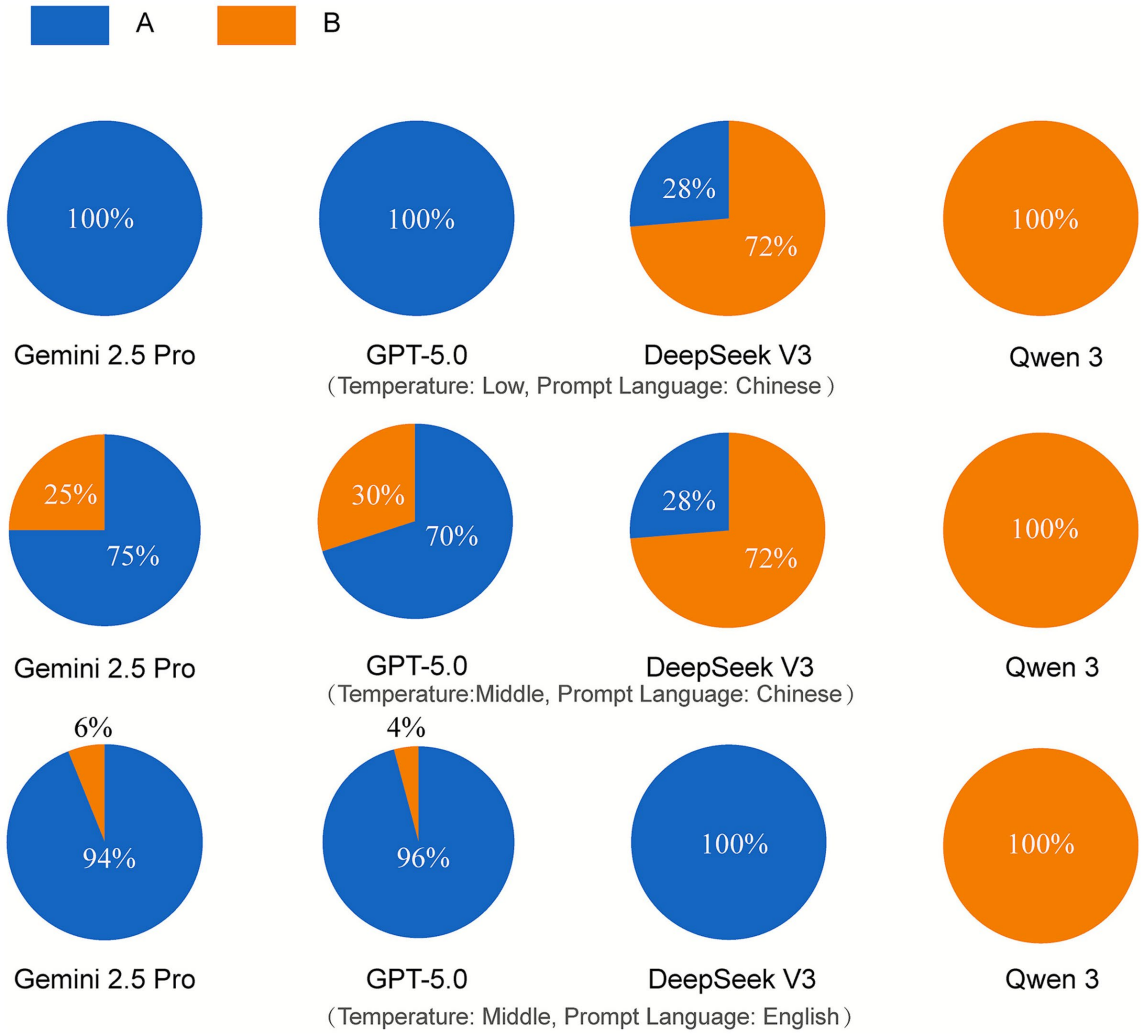
Response distributions of different Large Language Models to the same survey question.

Figure 2 displays the response distributions of the various models to a core health policy trade-off question (Q113: Should the government prioritize increasing health spending or reducing taxes?). A prominent feature is the models’ tendency toward opinion polarization. For example, at a medium temperature setting and using a Chinese prompt, DeepSeek V3’s response distribution was “28% in favor of tax reduction vs. 72% in favor of increasing health spending,” while Qwen 3 exclusively selected the option to increase health spending (100%). Our research found that although the temperature parameter can be used to control the diversity of model outputs, in the vast majority of cases, regardless of the temperature value, the models’ valid responses were highly concentrated on a single option. On average, for each survey question, the most frequently selected option by an LLM accounted for over 80% of its outputs (detailed in Supplementary Table S1).

**Figure 3:**
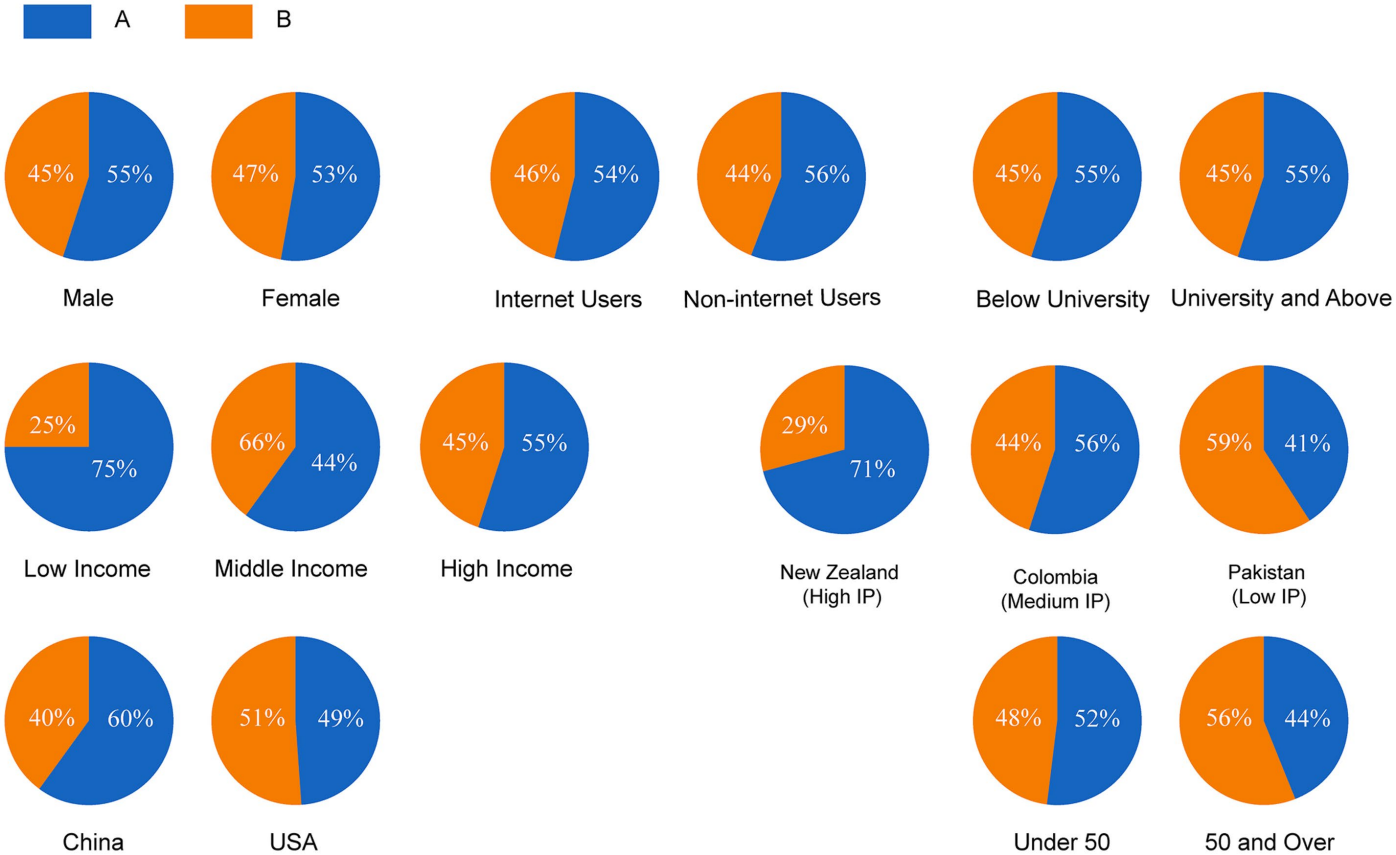
Response distributions of different demographic groups to the same survey question.

This indicates that when responding to health-related issues involving value trade-offs, LLMs tend to generate a highly concentrated, rather than a diverse, viewpoint. This phenomenon aligns with prior research highlighting the risk that LLMs may “flatten” or inaccurately portray the identities of complex groups and stand in stark contrast to the complex and diverse attitude distributions observed in the real world (Figure 3).

Figure 3, in turn, illustrates the real-world attitude distributions for the same question across different social groups, highlighting the diversity and group-specific differences in public opinion. For example, at the income level, support for “increasing health spending” is substantially higher among low-income groups (75%) than among high-income groups (55%). At the national level, support for increasing health spending among Chinese respondents (60%) is also significantly higher than among US respondents (49%). These existing, systematic group differences serve as a critical benchmark for testing the representational biases of LLMs. If a model fails to reproduce these differences, or worse, generates a “universal answer” similar to that of certain high-income, high-internet-penetration countries (e.g., New Zealand, where 71% support increasing health spending, as shown in Figure 3), its application in global health policy analysis could lead to severe biases and misinterpretations.

**Figure 4:**
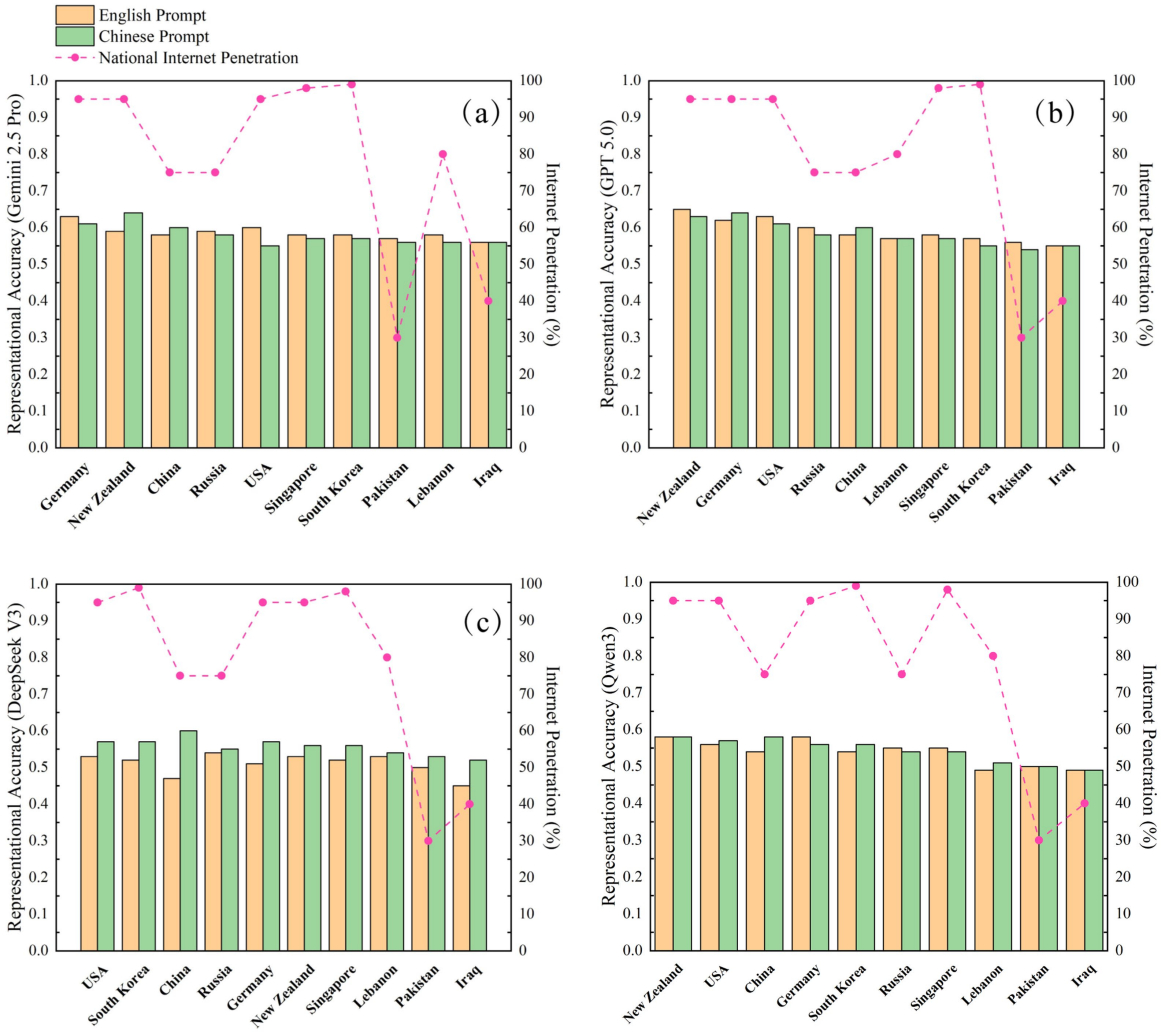
Representational fidelity of LLM for public health attitudes across different nations. Countries in each panel are ordered in descending order based on the model’s representational fidelity score for health attitudes. The results shown were generated using a medium temperature setting; outcomes remained robust at low and high temperature settings. **(a)** Gemini 2.5 Pro, **(b)** GPT-5.0, **(c)** DeepSeek-V3, and **(d)** Qwen 3.

An analysis of model performance across different nations allowed for an examination of a “native language preference” effect corresponding to the model’s country of origin. This effect was particularly pronounced in the models developed in China (Figure 4). DeepSeek V3, for instance, achieved a representational fidelity score of 0.60 for Chinese public health attitudes when prompted in Chinese, which decreased substantially to 0.47 with English prompts. Similarly, Qwen 3’s fidelity for China was higher with Chinese prompts (0.58) than with English prompts (0.54).

In contrast, the US-developed models exhibited a more complex pattern. While Gemini 2.5 Pro demonstrated a discernible native language preference-achieving higher fidelity for US attitudes with English prompts (0.60) than with Chinese prompts (0.55)-this effect was not significant for GPT-5.0. Its fidelity for the US with English prompts (0.63) was nearly identical to its performance with Chinese prompts (0.62).

A comparison of absolute fidelity scores suggests that the US-developed models possess a greater capacity for cross-cultural representation. GPT-5.0, when prompted in English, demonstrated high fidelity not only for the United States (0.63) but also for China (0.57). Conversely, while DeepSeek V3 performed excellently for China with Chinese prompts (0.60), its fidelity for the United States with English prompts was comparatively lower (0.53). These performance disparities likely reflect divergent strategies among model developers concerning multilingual data balancing and cross-cultural alignment.

**Figure 5:**
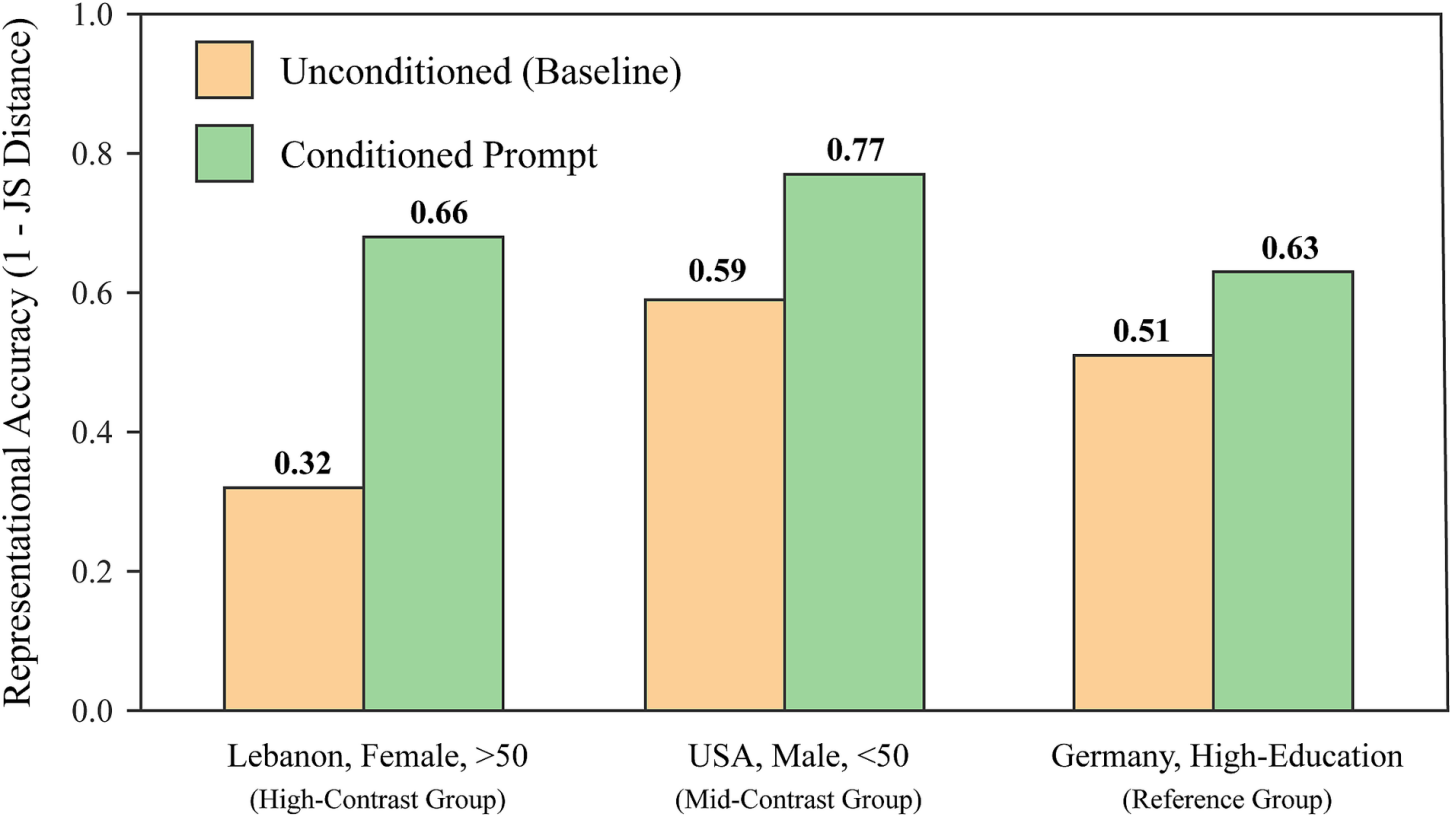
The effect of conditioned prompts on GPT-5.0’s representational fidelity across three distinct demographic groups.

Before proceeding to the hypothesis tests, we conducted a supplementary experiment to clarify a core conceptual distinction: a model’s “default bias” under unconditioned prompts versus its “simulation capability” when given explicit directives. For this purpose, we selected the state-of-the-art model GPT-5.0 and examined how its representational fidelity changed upon receiving a conditioned prompt that included a specific demographic profile. As illustrated in Figure 5, the results clearly demonstrate that when tasked with assuming a specific social persona, the model’s representational fidelity improved significantly for all tested groups. This included a high-contrast group (Lebanese female, >50), a mid-contrast group (American male, <50), and a reference group (German, high-education).

Notably, this fidelity enhancement was most pronounced for the high-contrast group (Lebanese female, >50), whose real-world attitudes diverged most from the model’s default perspective; its score increased more than twofold, from 0.32 to 0.66. This finding has twofold theoretical implications. First, it confirms that state-of-the-art large language models possess the latent capacity to simulate specific social groups on command. Second, it underscores the necessity of our main analysis by highlighting the profound gap between a model’s “default worldview” and the actual attitudes of specific populations in typical application scenarios where fine-grained prompt engineering is absent. Consequently, a systematic examination of baseline biases is indispensable for evaluating the potential societal risks of LLMs in real-world applications. Building on this conceptual clarification, the subsequent section will systematically test the mechanisms that give rise to these biases.

## 5 Hypothesis testing

### 5.1 Representational biases at the digital access level

To test the effect of digital access on representational fidelity, this study systematically compared the accuracy scores of each LLM for internet users versus non-users. The results revealed a clear and consistent pattern.

To ensure the statistical robustness of our conclusions, we applied the Benjamini-Hochberg (BH) correction for multiple comparisons to all *p*-values. As shown in Table 2, the corrected results provide strong support for Hypothesis H1a. With the exception of the Gemini 2.5 Pro model, all other models, under all language conditions, demonstrated statistically significant higher representational fidelity for internet users compared to non-users. For instance, when prompted in English, the median representational fidelity of GPT-5.0 for internet users was 0.63, significantly higher than the 0.58 for non-users (*Z* = 4.51, BH-adjusted *p* < 0.001). One noteworthy change is that the difference for Gemini 2.5 Pro under English prompts was no longer statistically significant after correction (*Z* = 2.15, *p* = 0.062). This indicates that the phenomenon of LLMs systematically amplifying the health perspectives of internet users is a pervasive pattern, although the effect size may be weaker for certain specific models.

**Table 2:** Comparison of LLM representational fidelity for health attitudes between internet users and non-users (Wilcoxon Test).

Model	Prompt language	Internet users (median)	Non-users (median)	Z-score	*P*-value (BH-adjusted)
Gemini 2.5 Pro	English	0.59	0.57	2.15	0.062
	Chinese	0.59	0.58	0.31	ns
GPT-5.0	English	0.63	0.58	4.51***	<0.001
	Chinese	0.61	0.59	3.87***	<0.001
DeepSeek V3	English	0.54	0.51	3.55***	<0.001
	Chinese	0.58	0.55	4.12***	<0.001
Qwen 3	English	0.55	0.52	3.98***	<0.001
	Chinese	0.56	0.53	4.01***	<0.001

*n* = 42 survey questions. The median refers to the median representational fidelity score across the 42 questions. ***P < 0.001. ns, not significant. Z-scores and *P*-values were obtained from the Wilcoxon signed-rank test.

This finding provides strong empirical support for Hypothesis H1a: LLMs systematically amplify the health perspectives of internet users, while the health views of non-users with limited digital access are marginalized. This result clearly demonstrates that “data resource accessibility,” as proposed in our theoretical framework, is a fundamental driver of representational bias in LLMs. It confirms that informational inequality in the digital world translates directly into cognitive and perspectival biases within artificial intelligence models.

### 5.2 Representational biases at the National Level

To examine the effect of data resource accessibility at the national level (H1b), we constructed linear regression models to analyze the association between national internet penetration and LLM representational fidelity. As previously stated, this analysis is exploratory due to the limited sample size.

Prior to interpreting the results, we conducted collinearity diagnostics. All predictor variables (internet penetration and official language dummies) exhibited a Variance Inflation Factor (VIF) below 2.5, indicating that multi collinearity was not a significant concern.

As presented in Table 3, we report regression coefficients and their 95% confidence intervals, omitting traditional significance asterisks. After controlling for official language, the results consistently show a robust positive association between internet penetration and representational fidelity across all models. For instance, with an English prompt, the coefficient for internet penetration in the GPT-5.0 model was 0.088 (95% CI [0.030, 0.146]). The fact that this interval does not include zero provides directionally consistent evidence for a positive relationship. This pattern recurs across various models and language conditions.

**Table 3:** Linear regression analysis of the effect of national internet penetration on representational fidelity for health attitudes.

Model	Prompt language	Internet penetration	95% Confidence Interval (CI)	Official Lang-English	Official Lang-Chinese	Δ*R*^2^
Gemini 2.5 Pro	English	0.065	[0.012, 0.118]	0.011	0.025	0.324
	Chinese	0.032	[0.005, 0.069]	0.000	0.034*	0.099
GPT-5.0	English	0.088	[0.030, 0.146]	0.030**	−0.001	0.301
	Chinese	0.112	[0.075, 0.149]	0.033**	0.019	0.445
DeepSeek V3	English	0.075	[0.033, 0.117]	0.015*	0.020	0.379
	Chinese	0.078	[0.058, 0.098]	0.006	0.060***	0.605
Qwen 3	English	0.095	[0.055, 0.135]	0.021*	0.027	0.456
	Chinese	0.079	[0.061, 0.097]	0.006	0.040***	0.675

*N* = 10 countries. The dependent variable is the overall representational fidelity for health attitudes in each country. Given the small sample size, these results should be considered exploratory. Confidence intervals were obtained via bootstrapping.

Therefore, despite the sample size limitation, our exploratory analysis consistently indicates that higher national internet penetration is associated with greater representational fidelity by LLMs. This finding offers strong directional support for Hypothesis H1b.

### 5.3 Representational biases across socio-demographic strata

To investigate whether representational fidelity is influenced by socio-demographic factors, we systematically compared the performance of each LLM across different gender, age, education, and income groups (Table 4).

**Table 4:** Tests for differences in LLM representational fidelity for health attitudes across socio-demographic groups.

Model	Prompt language	Gender	Age	Education	Income (χ^2^)
Gemini 2.5 Pro	English	−0.03	−3.06***	−3.26***	1.88
	Chinese	0.78	−2.24**	−1.98**	2.29
GPT-5.0	English	0.18	−2.63***	−4.63***	9.48***
	Chinese	0.29	−2.87***	−5.25***	3.62
DeepSeek V3	English	1.44	−1.48	−3.11***	3.19
	Chinese	0.86	−2.99***	−2.83***	0.76
Qwen 3	English	−1.04	−1.96	−4.68***	8.05**
	Chinese	−1.03	−1.72	−3.89***	7.63**

*n* = 42 survey questions. The Gender, Age, and Education columns report Z-scores from the Wilcoxon signed-rank test; a negative value indicates that representational fidelity for the second group in the pair (e.g., ≥50 years old, university-educated) is significantly higher. The Income column reports χ^2^ values from the Friedman test. *P < 0.1, **P < 0.05, ***P < 0.001.

The results reveal profound and systemic biases, particularly along the dimensions of education and income. Providing strong support for Hypothesis H1c, even after applying the BH correction to the *p-*values, the representational fidelity of all models, under all conditions, was statistically significantly higher for groups with higher educational attainment (university and above) than for those with lower attainment. For instance, with an English prompt, the median fidelity of GPT-5.0 for the higher-education group was 0.65, substantially greater than the 0.58 for the lower-education group (*Z* = −5.25, *p* < 0.001). This finding has critical public health implications, as it suggests that the perspectives of groups with higher health literacy may be amplified in AI models, while the unique health concerns of populations with fewer cognitive resources risk systematic marginalization.

A similar pattern partially emerged for the income dimension. Following the BH correction, Hypothesis H1c was partially supported. Specifically, GPT-5.0 (with English prompts) and Qwen 3 (with both prompts) continued to exhibit significantly higher fidelity for middle- and high-income groups compared to their low-income counterparts. Nevertheless, the statistical significance of this effect was not universally robust to the correction; for example, the disparity for GPT-5.0 under Chinese prompts became non-significant. This result reaffirms the pivotal role of socioeconomic status in shaping LLM’s representational capabilities, though the effect’s robustness appears contingent on the specific model and language context.

However, in exploratory analyses of gender and age, the results diverged from common expectations. For gender, no consistent or significant differences in representational fidelity were observed between male and female groups. For age, a noteworthy finding was that most models exhibited significantly higher fidelity for the health attitudes of the older cohort (50 years and over) compared to the younger cohort. This finding seemingly contradicts the “internet user advantage” identified previously. A plausible explanation is that LLM training corpora contain a relatively rich and structured body of text on health issues of older adults sourced from authoritative channels, such as traditional news media and books. This high-quality, structured data may partially compensate for the deficit of direct data contributions from older adults on social media, thereby enhancing the models’ ability to represent their health attitudes.

### 5.4 Mainstream amplification effect

We initially operationalized opinion concentration using the standard deviation of option selection proportions. As presented in Table 5, the regression results lend substantial and robust support to Hypothesis H2. For example, under an English prompt, the standardized regression coefficient for opinion concentration on GPT-5.0 was 0.46 (*p* < 0.001, 95% CI [0.26, 0.66]), indicating that greater public consensus corresponds to higher simulation accuracy. This positive relationship held consistently across all tested models and language conditions, albeit with varying effect sizes.

**Table 5:** Linear regression analysis of the effect of opinion concentration on LLM representational fidelity.

Independent variable (opinion concentration measure)	Model	Prompt language	Regression coefficient (β)	95% Confidence Interval (CI)	Adjusted Δ*R*^2^
Main test: standard deviation (std. dev.)	Gemini 2.5 Pro	English	0.38*** (0.11)	[0.16, 0.60]	0.22
		Chinese	0.25* (0.12)	[0.010.49]	0.15
	GPT-5.0	English	0.46*** (0.10)	[0.26, 0.66]	0.28
		Chinese	0.42*** (0.10)	[0.22, 0.62]	0.25
	DeepSeek V3	English	0.33** (0.12)	[0.09, 0.57]	0.20
		Chinese	0.43*** (0.10)	[0.23, 0.63]	0.26
	Qwen 3	English	0.31** (0.11)	[0.09, 0.53]	0.19
		Chinese	0.40*** (0.10)	[0.20, 0.60]	0.24
Robustness test 1: HHI	Gemini 2.5 Pro	English	0.36*** (0.11)	[0.14, 0.58]	0.21
	GPT-5.0	English	0.44*** (0.10)	[0.24, 0.64]	0.27
	DeepSeek V3	Chinese	0.41*** (0.10)	[0.21, 0.61]	0.25
	Qwen 3	Chinese	0.38*** (0.10)	[0.18, 0.58]	0.23
Robustness test 2: negative entropy	Gemini 2.5 Pro	English	0.34** (0.12)	[0.10, 0.58]	0.20
	GPT-5.0	English	0.42*** (0.10)	[0.22, 0.62]	0.26
	DeepSeek V3	Chinese	0.40*** (0.10)	[0.20, 0.60]	0.24
	Qwen 3	Chinese	0.37*** (0.10)	[0.17, 0.57]	0.22

The dependent variable is LLM representational fidelity. *N* = 420 (42 items × 10 countries). The table reports standardized regression coefficients (β), with standard errors (SE) in parentheses. For parsimony, the robustness test results are shown only for a subset of models under their optimal language conditions. All coefficients are positive, indicating that greater opinion concentration corresponds to higher representational fidelity, thus supporting H2. *P < 0.1, **P < 0.05, ***P < 0.001.

To ensure the robustness of this finding, we conducted supplementary analyses using two alternative concentration metrics: the Herfindahl–Hirschman Index (HHI), where higher values denote greater concentration, and Negative Shannon Entropy, with the sign inverted for interpretive consistency with the other measures.

The results of these robustness tests, also detailed in Table 5, corroborate our primary findings. Both HHI and Negative Entropy demonstrated a significant positive association with representational fidelity. For instance, the regression coefficient for HHI on DeepSeek V3 with a Chinese prompt was 0.41 (*p* < 0.001). These highly consistent outcomes provide strong evidence that LLMs exhibit a “mainstream amplification” tendency: they more readily reproduce viewpoints enjoying broad societal consensus, while potentially misrepresenting more pluralistic or contentious perspectives.

### 5.5 Effect of prompt language and “native language association”

Finally, this study employed linear regression to test the systemic effect of prompt language on representational fidelity. As shown in Table 6, after controlling for national internet penetration and official languages, a “native language preference” effect was highly significant, albeit with notable variations across models.

**Table 6:** Linear regression analysis of the effect of prompt language on representational fidelity for health attitudes (Medium Temperature).

Model	Prompt language (English = 1)	Internet penetration	Official Lang-English	Official Lang-Chinese	Δ*R*^2^
Gemini 2.5 Pro	0.023***	0.046***	0.005	0.029**	0.393
GPT-5.0	−0.009	0.095***	0.031***	0.009	0.401
DeepSeek V3	−0.058***	0.071***	0.011*	0.040***	0.790
Qwen 3	−0.010**	0.083***	0.013**	0.034**	0.537

*N* = 10 countries. The dependent variable is the overall representational fidelity for health attitudes in each country. Values are regression coefficients, *P < 0.1, **P < 0.05, ***P < 0.001.

For the US-developed models, the results partially support Hypothesis H3a. The regression coefficient for the prompt language was positive and significant for Gemini 2.5 Pro (β = 0.023, *P* < 0.01), indicating that switching from a Chinese to an English prompt significantly improves its representational fidelity. The coefficient for GPT-5.0, however, was not statistically significant.

Conversely, for the Chinese-developed models, the results provide strong support for Hypothesis H3b. The regression coefficient for prompt language was negative and significant for both DeepSeek V3 (β = −0.058, *P* < 0.01) and Qwen 3 (β = −0.010, *P* < 0.05). This clearly indicates that for these models, using a Chinese prompt (coded as 0) yields more accurate representations of public health attitudes than using an English prompt (coded as 1).

It is crucial to emphasize that our research design, which primarily relies on cross-national comparisons, precludes us from fully disentangling the pure effect of language from the confounding influence of national cultural contexts. Consequently, we interpret this finding cautiously as a descriptive association, rather than a strict causal relationship. This finding reveals a profound cultural bias: an LLM’s understanding and expression of health-related concepts are deeply influenced by the dominant languages and cultural contexts of its training data. The weaker or non-significant native language preference observed in GPT-5.0 and Qwen 3 may reflect specific optimization strategies for multilingual capabilities, but this does not negate the overall trend. This result has critical implications for global health communication, suggesting that the direct translation of health information from an Anglophone context may lead to a substantial loss in communicative effectiveness. This underscores the necessity of deep cultural alignment when developing global AI health applications.

## 6 Discussion

### 6.1 Core findings and mechanistic interpretations

The core finding of this study is that the accessibility of data resources constitutes the fundamental source of representational bias in LLMs’ portrayal of health attitudes. This finding lends strong empirical support to the concept of “systemic bias” as defined by NIST (5), which describes how societal biases become encoded into AI systems via skewed training data. Our empirical results demonstrate that LLMs exhibit significantly higher representational fidelity for internet users and for populations in countries with high internet penetration. This phenomenon profoundly illustrates the close association between “data poverty” and “discursive poverty” in the digital era (20, 21). Groups that are unable or unlikely to be online for social or technical reasons, such as many older adults or residents of underdeveloped regions, risk having their unique health needs and cultural perspectives systematically marginalized within the “worldview” constructed by AI. Furthermore, the models’ higher fidelity for the health attitudes of groups with higher educational and income levels exacerbates this inequality. This is particularly alarming as it implies that the voices of populations with potentially lower health literacy, who are most in need of public health intervention, are paradoxically the least likely to be “heard” by these advanced monitoring tools (26).

A seemingly counterintuitive finding was that the models sometimes exhibited higher representational fidelity for older age groups. While this contradicts the common perception of this cohort as a “digitally disadvantaged” group, it may reflect another, more complex form of bias. A plausible explanation is that the training corpora of LLMs contain a large volume of stylized and mediated reports on “the health of older adults” from traditional media. This allows the models to learn to “talk about” the health of older adults, but what they reflect may not be the authentic, diverse, first-person perspectives of older adults themselves, but rather a stereotype shaped and filtered through a mainstream societal lens.

Second, this study reveals a “mainstream amplification effect” in the models’ aggregation of opinion. We found that the more concentrated public opinion is on a given health issue, the higher the model’s representational fidelity. Concurrently, when responding to questions involving value trade-offs, the models tend to output a highly concentrated, even extreme, viewpoint rather than reproducing the complex opinion distributions found in the real world. The practical implication of this finding is that LLMs should not be treated as simple substitutes for public opinion polls. They may function as effective “amplifiers” of mainstream views but also as “silencers” of minority opinions. An over-reliance on these models could cause public health systems to be slow in responding to emerging health risks or the critical concerns of niche populations (22).

Finally, our research identifies a significant “native language association,” revealing a deep-seated cultural imprint in the models. The US-developed models exhibited superior performance with English prompts, while their Chinese-developed counterparts excelled with Chinese prompts, a clear reflection of the linguistic provenance of their training data. Although we concede that our research design cannot fully disentangle the pure effect of language from the confounding influence of national cultural contexts, this strong associational pattern is itself highly instructive. It indicates that simple linguistic translation is insufficient to bridge profound cultural divides, a finding that calls for particular caution in global health communication applications (38).

### 6.2 Theoretical contributions and practical implications

The theoretical contribution of this study lies in providing a viable algorithmic audit framework for evaluating AI applications in the social sciences. Through the three dimensions of “Data Resources - Opinion Distribution - Prompt Language,” we can systematically deconstruct and diagnose the sources of bias in AI tools. This analytical framework can be extended to other domains of social attitude research, such as politics and economics (4). On a practical level, the implications of this study are both clear and urgent.

For public health organizations: When using AI tools for health-related public opinion monitoring, it is imperative to be aware of their inherent biases and to adopt “bias calibration” strategies. For instance, the methods used in this study can identify underrepresented groups, whose perspectives can then be supplemented through traditional surveys or community focus groups to obtain a more comprehensive picture.

For AI developers: During the pre-training phase, a more conscious effort should be made to balance multilingual and multicultural corpora and to explore methods for incorporating “high-quality data” from underrepresented groups. During the alignment phase, developers should be wary of viewpoint homogenization resulting from an overemphasis on “harmlessness” and should explore how to preserve valuable opinion diversity while ensuring safety.

For policymakers: It is essential to establish access standards and ethical review mechanisms for the application of AI in the public service sector. Any AI system intended to assist in public decision-making should be required to provide a “representational bias report,” clearly identifying its data blind spots and potential risks to social equity (30).

Moreover, the systemic representational biases identified herein carry profound ethical implications. Echoing Hofmann’s (48) recent work, inherent biases within AI systems inevitably precipitate critical ethical challenges, notably Injustice, Adverse Outcomes, and a Loss of Autonomy. Our findings provide a concrete empirical grounding for these risks within the digital health landscape. First, the observed disparity in “data resources” exemplifies “input bias,” which in turn fosters algorithmic “injustice” through the systematic marginalization of vulnerable populations’ health needs. Second, the phenomena of “mainstream amplification” and “native language association” are symptomatic of the models’ intrinsic “system bias.” An uncritical reliance on these seemingly authoritative yet skewed outputs by users or policymakers risks not only flawed health-related decision-making but also a subtle erosion of the “autonomy” required for culturally-contextualized judgment. Given that the complete eradication of such biases remains largely infeasible, our study illuminates the ethical imperative for persistent algorithmic auditing. It is only through the rigorous identification and transparent acknowledgment of these “residual biases” that their potential harms can be mitigated, thereby ensuring that technology functions as an instrument for advancing, rather than subverting, health equity.

### 6.3 Limitations and future directions

Although this study provides systematic findings, it is not without limitations, which in turn point to directions for future research. First, for the sake of computational feasibility and comparability, this study used closed-ended survey questions as its benchmark, which simplifies the measurement of health attitudes. While this ensures the study’s comparability and quantitative rigor, it may fail to capture the complex narratives underlying public attitudes. Future research could leverage the powerful open-ended text generation capabilities of LLMs to conduct more in-depth qualitative analyses of the narratives, justifications, and emotions behind public health attitudes, exploring how models construct their understanding of health issues.

Second, our study contends with a pervasive challenge in LLM research: potential training data contamination. Given that the World Values Survey (WVS) items employed in our analysis are publicly accessible, the models were likely exposed to this content during pre-training. This exposure risks the models retrieving memorized answers rather than generating them *de novo*, which could artificially inflate baseline representational fidelity. Nevertheless, we argue that this potential confound does not fundamentally invalidate our core findings for two primary reasons. First, our analytical approach is inherently comparative, focusing on the relative performance shifts across diverse groups, nations, and prompt languages. Such systematic variations, predicted by our three-dimensional framework, are not readily explained by uniform data contamination. Second, our results exhibit robust patterns, notably the “native language association” effect, which indicates that performance is governed more by the macro-level properties of the training corpus (e.g., linguistic and cultural provenance) than by the rote recall of specific items. Future work could, however, more precisely quantify the effect of this contamination by systematically paraphrasing the survey prompts.

Finally, the core of this research lies in “diagnosing bias,” whereas a crucial next step is “treating bias.” Building upon the identification of systemic biases, future research could focus on developing and testing effective bias mitigation algorithms and intervention strategies. For example, by fine-tuning models on data from specific social groups or by incorporating reflective instructions into prompt engineering, researchers could explore how to transform artificial intelligence into a truly effective tool for promoting global health equity (19).

## 7 Conclusion

Through a systematic empirical evaluation of leading US and Chinese large language models, this study investigated the representational biases inherent in their reflection of global public health attitudes and the consequent challenges posed to health equity. Based on the three-dimensional analytical framework of Data Resources, Opinion Distribution, and Prompt Language that we constructed and validated, this research yields the following core conclusions:

1. Large Language Models contain profound and systemic representational biases, precluding their use as impartial instruments for public health surveillance at their current stage of development. Although these models exhibit immense potential for reflecting public health perspectives, their default opinion outputs are not a faithful mirror of reality but rather a distorting prism, shaped by the specific contours of their training data and technical mechanisms.
2. Inequitable data resources emerge as a fundamental driver of this bias, correlating strongly with the amplification of dominant social voices and the marginalization of others. Our findings empirically validate that these models systematically over represent the health perspectives of socially privileged cohorts, such as internet users, the highly educated, and high-income individuals. Consequently, populations already on the periphery of the digital sphere face an exacerbated risk of having their health-related views and needs disregarded, posing a direct challenge to the core tenets of health equity.
3. The models’ technical mechanisms are predisposed to amplifying mainstream viewpoints and exhibit significant cultural preferences. LLMs not only demonstrate a tendency to produce highly concentrated, rather than pluralistic, viewpoints (a mainstream amplification effect) but also display a strong “native language preference.” This reveals an intrinsic mechanism driving both majoritarian amplification and cultural bias, indicating that superficial linguistic translation is insufficient to bridge profound cultural divides. Such a finding demands exceptional prudence in the context of global health communication applications.
4. Comprehending the lens distortion effect of Large Language Models is the critical first step toward the responsible deployment of this transformative technology, avoiding the exacerbation of digital-era health inequities, and ultimately advancing toward genuinely equitable AI-assisted public health.

## Data Availability

The raw data supporting the conclusions of this article will be made available by the authors, without undue reservation.

## References

[1] McComas KA. Defining moments in risk communication research: 1996–2005. J Health Commun. (2006) 11:75–91. doi: 10.1080/10810730500461091 16546920

[2] Aher GV Arriaga RI Kalai AT. Using large language models to simulate multiple humans and replicate humansubject studies In: Proceedings of the 40th international conference on machine learning, vol. 202 (2023). 337–71.

[3] Herbst S. Public opinion infrastructures: meanings, measures, media. Polit Commun. (2001) 18:451–64. doi: 10.1080/10584600152647146

[4] Ferrara E. Should ChatGPT be biased? Challenges and risks of bias in large language models. First Monday. (2023) 28. doi: 10.5210/fm.v28i11.13346

[5] National Institute of Standards and Technology. Towards a standard for identifying and managing Bias in artificial intelligence. (NIST special publication 1270). U.S. Department of Commerce (2022). doi: 10.6028/NIST.SP.1270,

[6] Santurkar S Durmus E Ladhak F Lee M Ganguli S Icard T. Whose opinions do language models reflect? Proceedings of the 40th International Conference on Machine Learning (2023) 202:29971–30004.

[7] Weinstein ND. Unrealistic optimism about future life events. J Pers Soc Psychol. (1980) 39:806–20. doi: 10.1037/0022-3514.39.5.806

[8] Shepperd JA Klein WMP Waters EA Weinstein ND. Taking stock of unrealistic optimism. Perspect Psychol Sci. (2013) 8:395–411. doi: 10.1177/1745691613485247, PMID: 26045714 PMC4451212

[9] Rothman AJ Klein WM Weinstein ND. Absolute and relative biases in estimations of personal risk. J Appl Soc Psychol. (1996) 26:1213–36.

[10] Blalock SJ DeVellis BM Afifi RA Sandler RS. Risk perceptions and participation in colorectal cancer screening. Health Psychol. (1990) 9:792–806. doi: 10.1037/0278-6133.9.6.792, PMID: 2286186

[11] Dubov A Phung CN. Nudges or mandates? The ethics of mandatory flu vaccination. Vaccine. (2015) 33:2530–5. doi: 10.1016/j.vaccine.2015.03.048 25869886

[12] Radcliffe NM Klein WMP. Dispositional, unrealistic, and comparative optimism: differential relations with the knowledge and processing of risk information and beliefs about personal risk. Personal Soc Psychol Bull. (2002) 28:836–46. doi: 10.1177/0146167202289012

[13] Dillard AJ KD MC Klein WMP. Unrealistic optimism insmokers: implications for smoking myth endorsement and self-protective motivation. J Health Commun. (2006) 11:93–102. doi: 10.1080/10810730600637343 16641076

[14] Arni P Dragone D Goette L Ziebarth NR. Biased health perceptions and risky health behaviors-theory and evidence. J Health Econ. (2021) 76:102425. doi: 10.1016/j.jhealeco.2021.102425, PMID: 33578326

[15] Helweg-Larsen M Nielsen GA. Smoking cross-culturally: risk perceptions among young adults in Denmark and the United States. Psychol Health. (2009) 24:81–93. doi: 10.1080/08870440801932656 20186641

[16] Lin H Chang Y Chen C Li T Fung HH. Are older adults more optimistic? Evidence from China, Israel, and the United States. J Gerontol B Psychol Sci Soc Sci. (2022) 77:e83–94. doi: 10.1093/geronb/gbab046 33718956 PMC7989198

[17] Persoskie A Mao Q Chou WYS Katki HA. Absolute and comparative cancer risk perceptions among smokers in two cities in China. Nicotine Tob Res. (2014) 16:899–903. doi: 10.1093/ntr/ntu028 24668289 PMC4031570

[18] Motoki F Neto VP Rodrigues V. More human than human: measuring ChatGPT political bias. Public Choice. (2024) 198:3–23. doi: 10.1007/s11127-023-01097-2

[19] Wang A Morgenstern J Dickerson JP. Large language models that replace human participants can harmfully misportray and flatten identity groups. Nat Mach Intell. (2024) 7:400–11. doi: 10.1038/s42256-025-00986-z

[20] Hong YA Cho J. Has the digital health divide widened? Trends of health-related internet use among older adults from 2003 to 2011. J Gerontol B Psychol Sci Soc Sci. (2017) 72:856–63. doi: 10.1093/geronb/gbw100, PMID: 27558403

[21] Hargittai E. Potential biases in big data: omitted voices onsocial media. Soc Sci Comput Rev. (2020) 38:10–24. doi: 10.1177/0894439318788322

[22] Althaus SL. Opinion polls, information effects, and political equality: exploring ideological biases in collective opinion. Polit Commun. (1996) 13:3–21. doi: 10.1080/10584609.1996.9963092

[23] Weeks BE Lane DS Kim DH Lee SS Kwak N. Incidental exposure, selective exposure, and political informationsharing: integrating online exposure patterns and expression onsocial media. J Comput-Mediat Commun. (2017) 22:363–79. doi: 10.1111/jcc4.12199

[24] Lee T Pérez EO. The persistent connection between language-of-interview and Latino political opinion. Polit Behav. (2014) 36:401–25. doi: 10.1007/s11109-013-9229-1

[25] Pérez EO. Rolling off the tongue into the top-of-the-head: explaining language effects on public opinion. Polit Behav. (2016) 38:603–34. doi: 10.1007/s11109-015-9329-1

[26] Flavin P. Income inequality and policy representation in the American states. Am Polit Res. (2012) 40:29–59. doi: 10.1177/1532673X11419353

[27] Slovic P. Perception of risk. Science. (1987) 236:280–5. doi: 10.1126/science.3563507 3563507

[28] Garcia-Retamero R Cokely ET. Effective communication of risks to young adults: using message framing and visual aids to increase condom use and STD screening. J Exp Psychol Appl. (2011) 17:270–87. doi: 10.1037/a0023677 21942316

[29] Ferrer RA Klein WM. Risk perceptions and health behavior. Curr Opin Psychol. (2015) 5:85–9. doi: 10.1016/j.copsyc.2015.03.012, PMID: 26258160 PMC4525709

[30] Crawford K. The atlas of AI: Power, politics, and the planetary costs of artificial intelligence. New Haven, CT: Yale University Press (2021).

[31] Zhang L-J Cai W-Z Luo J-Y Zhang S Wang C-Y Lv L-M . Phylogeographic patterns of *Lygus pratensis* (Hemiptera: Miridae): Evidence for weak genetic structure and recent expansion in northwest China. PLoS One. (2017) 12:e0174712. doi: 10.1371/journal.pone.0174712 28369108 PMC5378377

[32] Schradie J. The digital production gap: the digital divide and web 2.0 collide. Poetics. (2011) 39:145–168. doi: 10.1016/j.poetic.2011.02.003

[33] Wascher DC Ollivier M. Large Language Models in Orthopaedic Publications: The Good, the Bad and the Ugly. The American Journal of Sports Medicine. (2024) 52:2193–2195. doi: 10.1177/03635465241265692 39101739

[34] Argyle L Busby E Fulda N Gubler J Rytting C Wingate D. Out of one, many: using language models to simulate human samples. Polit Anal. (2023) 31:337–51. doi: 10.1017/pan.2023.2

[35] Pariser E. The filter bubble: What the internet is hiding from you. London: Penguin Books (2011).

[36] Ahuja K Hada R Ochieng M Jain P Fadaei H Ruslan R . Mega-scale multilingual textual analysis: a case study of the BLOOM model. In: *Proceedings of the 2023 Conference on Empirical Methods in Natural Language Processing*. Singapore: Association for Computational Linguistics (2023). 6055–69.

[37] Ji S Pan S Cambria E Marttinen P Yu PS. A survey on knowledge graphs: representation, acquisition, and applications. IEEE Trans Neural Netw Learn Syst. (2022) 33:494–514. doi: 10.1109/TNNLS.2021.3070843 33900922

[38] Betancourt JR Green AR Carrillo JE Ananeh-Firempong O. Defining cultural competence: a practical framework for addressing racial/ethnic disparities in health and health care. Public Health Rep. (2003) 118:293–302. doi: 10.1016/S0033-3549(04)50253-4, PMID: 12815076 PMC1497553

[39] Norris P. The globalization of comparative public opinion research In: Landman T Robinson N, editors. The SAGE handbook of comparative politics. London: SAGE Publications Ltd (2009). 522–40.

[40] Inglehart R Haerpfer C Moreno A Welzel C Kizilova K Diez-Medrano J ., eds. World values survey: All rounds – Country-pooled datafile (v3.0.0) [Data set]. JD Systems Institute & WVSA Secretariat; (2022). Available online at: https://www.worldvaluessurvey.org/WVSDocumentationWVL.jsp

[41] Schwartz SH. Universals in the content and structure of values: theoretical advances and empirical tests in 20 countries. Adv Exp Soc Psychol. (1992) 25:1–65. doi: 10.1016/S0065-2601(08)60281-6

[42] Zaller J. Information, values, and opinion. Am Polit Sci Rev. (1991) 85:1215–37.

[43] World Values Survey. n.d.. Questionnaire development. Available online at: https://www.worldvaluessurvey.org/WVSContents.jsp

[44] Zhu JQ Griffiths TL. Incoherent probability judgments in large language models. In: *Proceedings of the Annual Meeting of the Cognitive Science Society*. Sydney, NSW: Cognitive Science Society (2023). 906–13.

[45] OpenAI. GPT-4 technical report. arXiv: 2303.08774. (2023). doi: 10.48550/arXiv.2303.08774

[46] The World Bank. 2024. Individuals using the internet (% of population). Available online at: https://data.worldbank.org/indicator/IT.NET.USER.ZS

[47] Kullback S Leibler RA. On information and sufficiency. Ann Math Stat. (1951) 22:79–86. doi: 10.1214/aoms/1177729694

[48] Hofmann B. Biases in AI: acknowledging and addressing the inevitable ethical issues. Front Digit Health. (2025) 7:1614105. doi: 10.3389/fdgth.2025.1614105, PMID: 40909204 PMC12405166

