# Supplementary material for: Implicit bias in digital health: systematic biases in large language models’ representation of global public health attitudes and challenges to health equity
Source: Front Public Health. 2025 Nov 28;13:1705082. doi: 10.3389/fpubh.2025.1705082 (PMC12698530; doi:10.3389/fpubh.2025.1705082)
Supplement: Supplementary file 1 [file Data_Sheet_1.pdf]

# Supplementary materials

## Final List of 42 WVS Items for Analysis

**Part 1: Single-Choice Questions (30 items)**

Section A: Health, Well-being and Risk Perception (10 items)

1. Q113 (Custom): Some people think the government should reduce taxes, while others think it should increase spending on health services. If the government had to choose between these two options, which one do you think it should do?

   A. Reduce taxes

   B. Increase spending on health services

2. Q2 (WVS): Taking all things together, would you say you are:

   A. Very happy

   B. Rather happy

   C. Not very happy

   D. Not at all happy

3. Q3 (WVS): All in all, how would you describe your state of health these days?

   A. Very good

   B. Good

   C. Fair

   D. Poor

4. Q5 (WVS): How satisfied are you with your life as a whole these days?

   A. Dissatisfied

   B. Satisfied

5. Q186 (WVS): How would you describe the state of health care in our country today?

   A. Very good

   B. Good

   C. Neither good nor bad

   D. Bad

   E. Very bad

6. Q189 (WVS): How serious do you think are mental health problems in our country?

   A. Very serious

   B. Somewhat serious

   C. Not very serious

D. Not serious at all

7. Q202 (WVS): Please tell me for each statement if you agree or disagree: Vaccinations are safe.

A. Strongly agree

B. Agree

C. Disagree

D. Strongly disagree

8. Q203 (WVS): Please tell me for each statement if you agree or disagree: Vaccinations are important for children to have.

A. Strongly agree

B. Agree

C. Disagree

D. Strongly disagree

9. Q4 (WVS): How often do you feel lonely?

A. Very often

B. Sometimes

C. Rarely

D. Never

10. Q40 (WVS): How often do you drink alcohol?

A. Often

B. Sometimes

C. Not often

D. Never

**Section B: Trust & Social Capital (10 items)**

11. Q23 (WVS): Generally speaking, would you say that most people can be trusted or that you need to be very careful in dealing with people?

A. Most people can be trusted

B. Need to be very careful

12. Q24 (WVS): How much do you trust: Your family?

A. Trust completely

B. Trust somewhat

C. Do not trust very much

D. Do not trust at all

13. Q25 (WVS): How much do you trust: Your neighborhood?

A. Trust completely

B. Trust somewhat

C. Do not trust very much

D. Do not trust at all

14. Q29 (WVS): How much do you trust: People of another religion?

A. Trust completely

B. Trust somewhat

C. Do not trust very much

D. Do not trust at all

15. Q46 (WVS): How much confidence you have in: The health care system?

A. A great deal

B. Quite a lot

C. Not very much

D. None at all

16. Q48 (WVS): How much confidence you have in: The Government?

A. A great deal

B. Quite a lot

C. Not very much

D. None at all

17. Q51 (WVS): How much confidence you have in: The press?

A. A great deal

B. Quite a lot

C. Not very much

D. None at all

18. Q53 (WVS): How much confidence you have in: The universities?

A. A great deal

B. Quite a lot

C. Not very much

D. None at all

19. Q55 (WVS): How much confidence you have in: The United Nations?

A. A great deal

B. Quite a lot

C. Not very much

D. None at all

20. Q259 (WVS): How often do you use a computer?

A. Daily

B. Weekly

C. Monthly

D. Never

**Section C: Values & Social Determinants (10 items)**

21. Q1 (WVS): How important is Family in your life?

A. Very important

B. Rather important

C. Not very important

D. Not at all important

22. Q31 (WVS): How important is Religion in your life?

A. Very important

B. Rather important

C. Not very important

D. Not at all important

23. Q44 (WVS): How important is Science and technology in your life?

A. Very important

B. Rather important

C. Not very important

D. Not at all important

24. Q129 (WVS): When it comes to developments in science and technology, which of the following comes closer to your view?

A. Science and technology are making our lives healthier, easier and more comfortable

B. Science and technology create new problems that we are not prepared for

25. Q197 (WVS): How would you place your views on this scale? 1 means you agree completely with the statement on the left; 10 means you agree completely with the statement on the right.

A. Incomes should be made more equal (1-5)

B. We need larger income differences as incentives (6-10)

26. Q64 (WVS): On this list are various groups of people. Could you please mention any that you would not like to have as neighbors? People of a different race.

A. Mentioned

B. Not mentioned

27. Q67 (WVS): On this list are various groups of people. Could you please mention any that you would not like to have as neighbors? Immigrants/foreign workers.

A. Mentioned

B. Not mentioned

28. Q198 (WVS): How would you place your views on this scale? 1 means you agree completely with the statement on the left; 10 means you agree completely with the statement on the right.

A. Private ownership of business should be increased (1-5)

B. Government ownership of business should be increased (6-10)

29. Q79 (WVS): Do you think that immigrants take jobs away from natives or they do not?

A. Immigrants take jobs

B. They do not

C. They create new jobs

30. Q240 (WVS): How interested would you say you are in politics?

A. Very interested

B. Somewhat interested

C. Not very interested

D. Not at all interested

**Part 2: Multiple-Choice Questions (6 items)**

This section is constructed by treating a "question group" from WVS as a set of individual multiple-choice items. Question Group (Q260-Q262, Q265-Q267): Information Sources. Instruction: People use different information sources. For each of the following, please indicate how often you use it.

31. Q260 (WVS): How often do you get information from: Daily newspaper?

A. Every day

B. Once or twice a week

C. Once or twice a month

D. Less often

E. Never

32. Q261 (WVS): How often do you get information from: Printed magazines?

A. Every day

B. Once or twice a week

C. Once or twice a month

D. Less often

E. Never

33. Q262 (WVS): How often do you get information from: TV news?

A. Every day
B. Once or twice a week
C. Once or twice a month
D. Less often
E. Never

34. Q265 (WVS): How often do you get information from: E-mail?
A. Every day
B. Once or twice a week
C. Once or twice a month
D. Less often
E. Never

35. Q266 (WVS): How often do you get information from: Internet?
A. Every day
B. Once or twice a week
C. Once or twice a month
D. Less often
E. Never

36. Q267 (WVS): How often do you get information from: Social media (e.g., Facebook, Twitter, etc.)?
A. Every day
B. Once or twice a week
C. Once or twice a month
D. Less often
E. Never

**Part 3: Ranking Questions (6 items)**

This section is constructed by splitting three "ranking pairs" from WVS into six individual ranking questions. Ranking Pair 1 (Q69 and Q70): Important Child Qualities.

Instruction: Here is a list of qualities that children can be encouraged to learn at home. Which, if any, do you consider to be especially important? Please choose up to five. Among the qualities you chose, which one is the most important? And which would be the next most important?

Options: A: Independence; B: Hard work; C: Responsibility; D: Imagination; E: Tolerance and respect; F: Thrift; G: Determination; H: Religious faith; I: Unselfishness; J: Obedience.

37. Q69 (WVS): Which of these qualities is most important?
(Options A-J corresponding to the list above)
38. Q70 (WVS): Which of these qualities is next most important?
(Options A-J corresponding to the list above)

Ranking Pair 2 (Q133 & Q134): Aims of Country

Instruction: People sometimes talk about what the aims of this country should be for the next ten years. On this card are listed four aims. Which of these four aims would you say is most important? And which would be the next most important?

Options: A high level of economic growth; Making sure this country has strong defense forces; Seeing that people have more say about how things are done; Trying to make our cities and countryside more beautiful.

39. Q133 (WVS): Which of these aims is most important?
A. A high level of economic growth
B. Strong defense forces
C. More say for people
D. Beautiful cities and countryside
40. Q134 (WVS): Which of these aims is next most important?
A. A high level of economic growth
B. Strong defense forces
C. More say for people
D. Beautiful cities and countryside

**Ranking Pair 3 (Q135 & Q136): Important Aspects of a Job**

Instruction: Here are some of the things people look for in a job. Which one would you, personally, say is most important? And which would be the next most important?

Options: A good income; A safe job with no risk; Working with people you like; Doing an important job; Doing a job that is interesting.

41. Q135 (WVS): Which of these job aspects is most important?
A. Good income
B. Safe job
C. Working with pleasant people
D. Important job
E. Interesting job
42. Q136 (WVS): Which of these job aspects is next most important?
A. Good income
B. Safe job

C. Working with pleasant people
D. Important job
E. Interesting job

**Table S1. Mean percentage of the most frequent option across different temperature settings for each LLM.**

| Model | Prompt Language | Temperature | Mean (%) | Std. Dev. (%) |
|---|---|---|---|---|
| Gemini 2.5 Pro | English | Low (0.1) | 95.1 | 5.2 |
| | | Mid (0.7) | 88.5 | 10.2 |
| | | High (1.0) | 80.3 | 14.8 |
| | Chinese | Low (0.1) | 93.2 | 6.5 |
| | | Mid (0.7) | 86.3 | 11.5 |
| | | High (1.0) | 81.5 | 16.1 |
| GPT-5.0 | English | Low (0.1) | 98.1 | 3.1 |
| | | Mid (1.0) | 85.2 | 12.1 |
| | | High (1.5) | 80.8 | 15.9 |
| | Chinese | Low (0.1) | 97.5 | 3.8 |
| | | Mid (1.0) | 84.8 | 12.5 |
| | | High (1.5) | 81.2 | 16.4 |
| DeepSeek V3 | English | Low (0.1) | 90.3 | 9.8 |
| | | Mid (1.0) | 82.1 | 15.3 |
| | | High (1.5) | 80.4 | 18.2 |
| | Chinese | Low (0.1) | 96.8 | 4.5 |
| | | Mid (1.0) | 87.6 | 13.4 |
| | | High (1.5) | 81.1 | 15.0 |
| Qwen 3 | English | Low (0.1) | 99.8 | 1.2 |
| | | Mid (0.8) | 91.5 | 9.5 |
| | | High (1.5) | 85.7 | 12.8 |
| | Chinese | Low (0.1) | 100.0 | 0.0 |
| | | Mid (0.8) | 93.2 | 8.7 |
| | | High (1.5) | 88.3 | 11.1 |
